# Alanine Mutagenesis in the Complementarity Determining Region 3 of the MTB and HIV-1 Peptide-Bispecific T Cell Receptor Beta Chain Affects Ligand Recognition

**DOI:** 10.3389/fimmu.2017.00983

**Published:** 2017-08-16

**Authors:** Chao-Ying Zhou, Rui-Ning Wang, Qian Wen, Wen-Ting He, Shi-Meng Zhang, Xia-Lin Du, Jia-Hui Yang, Li Ma

**Affiliations:** ^1^Institute of Molecular Immunology, School of Laboratory Medicine and Biotechnology, Southern Medical University, Guangzhou, China

**Keywords:** alanine scanning mutagenesis, complementarity determining region 3β, bispecific T cell receptor, MTB/HIV coinfection, adoptive immunotherapy

## Abstract

*Mycobacterium tuberculosis*/human immunodeficiency virus (MTB/HIV) coinfection presents a special challenge to the prevention and treatment of tuberculosis and HIV/AIDS. Adoptive transfer of high-affinity T cell receptor (TCR) gene-modified T cells against MTB and HIV antigens is a promising approach to treating MTB/HIV coinfected patients whose cellular immunity is obviously disordered. We have previously successfully identified that a bispecific TCR screened out from peripheral blood mononuclear cells of a HLA-A*0201^+^ healthy individual using the complementarity determining region 3 (CDR3) spectratype analysis recognizes both MTB Ag85B_199–207_ and HIV-1 Env_120–128_ peptide. However, it has not been known how residues on CDR3 loops, which have been shown to play a leading role in antigen binding and specificity contribute to the bispecific TCR contact with the peptide–major histocompatibility complex (MHC) complexes. In this study, we provided an extensive investigation of residues in the predicted CDR3 of the bispecific TCR beta (β) chain using alanine scanning mutagenesis. Our data showed that three of the five substituted residues (G115A, T116A, A117G) in CDR3β of the bispecific TCR caused a significantly diminished T cell response to antigen, whereas the remaining two substituted residues (D114A, S118A) resulted in completely eliminated response, thus identifying the two residues that were particularly critical for the recognition of peptide–MHC in the bispecific TCR. These findings will provide an imperative foundation for generating an improved high-affinity bispecific TCR for use in T cell adoptive immunotherapy for MTB/HIV coinfected individuals.

## Introduction

*Mycobacterium tuberculosis*/human immunodeficiency virus (MTB/HIV) coinfection presents a massive challenge to the prevention and treatment of tuberculosis (TB) and HIV/AIDS. HIV infection is the most powerful risk factor for development of active TB, which remarkably increases the individual susceptibility to primary TB infection or reinfection and promotes reactivation of latent TB infection (1). TB also has an adverse impact on immune responses to HIV, which accelerates the course of disease from HIV infection to AIDS (1). Among the estimated 10.4 million new TB cases in 2015, about 1.2 million (11%) were estimated to be HIV positive. There were an estimated 1.8 million TB deaths in 2015, among which about a quarter were people who were infected with HIV (2). Notwithstanding World Health Organization guidelines supporting long courses of concomitant anti-TB and antiretroviral therapies of the two diseases and urging more aggressive management, multiple problems such as potential drug interactions between rifampin and some kinds of antiretroviral drugs, cumulative drug toxicities, a high pill burden, the immune reconstitution inflammatory syndrome, and programmatic challenges still exist (3). Therefore, development of new therapeutic options for MTB/HIV coinfection has become imperative.

*Mycobacterium tuberculosis* and HIV are both intracellular pathogens that can be controlled by cellular immunity mediated by T cells. However, the immune function is obviously disordered in MTB/HIV coinfected patients, which mainly manifest as reduced number and declining function of effector T cells (4). Hence, cell-based immunotherapy based on increasing T cell numbers and improving T cell function is a promising approach for treating MTB/HIV coinfected patients. Adoptive T cell transfer has shown some prominent antitumor or anti-infection responses in patients with cancer and chronic infection (5–7). A main restriction of universal application of adoptive cellular immunotherapy is the difficulty in generating sufficient numbers of human T cells with antitumor or anti-infection potential. Rosenberg group has proved that about only half of melanomas reproducibly give rise to melanoma-specific lymphocyte (8). As an alternative method, high-affinity T cell receptors (TCRs) can be introduced into autologous T cells of the patients, and then transfer these antigen-specific TCR gene-modified T cells into lympho-depleted patients, which has shown notable therapeutic effect in treatment of metastatic melanoma (9), leukemia (10), hepatitis C-associated hepatocellular carcinoma (11), and HIV (12), cytomegalovirus (13), and Epstein–Barr virus infections (14).

Several lines of evidence suggest that TCR affinity is the primary factor for determining the avidity of T cells and the consequence of antigen stimulation (15, 16). The rigor of thymic positive and negative selection ensures that natural TCRs, which bind to widespread self or tumor-associated antigens possess virtually much lower affinities than when they bind to pathogen antigens (17). Native TCR–peptide–major histocompatibility complex (MHC) interactions have an extremely narrow window of affinities in the range of 0.1–500 µM (18), which possibly reflect a balance between the need to efficiently activate T cells and the need to sustain immunologic self-tolerance (19). Within this range, TCR affinity is associated with antigen sensitivity (20), which puts self-antigen-specific T cells at an obvious disadvantage in comparison with their pathogen-reactive T cells. The transfer of genes encoding TCRs with affinities as high as those of the best antiviral T cells (*K*_d_ = 100 nM) into recipient T cells could offer optimal anti-infection immunity (18). Because TCRs with higher affinity do not appear in the mature T cell repertoire, understanding the molecular basis of natural TCRs, which requires the mutational analysis, will establish a foundation for obtaining the engineered high-affinity TCRs.

Alanine scanning mutagenesis within the complementarity determining regions (CDRs) of the α- and β-chains, which form the TCR heterodimer is a commonly used method of mutational analysis to illuminate the function of TCR amino acid (AA) residues in antigen complex binding as well as activation of T cells (21). CDRs that engage both the peptide and the MHC molecule are surface-exposed loops, which play a dominant role in determining the specificity of antigen recognition by TCR. As is well known, TCRs and peptide–MHC interactions are a two-step process whereby the CDR1 and CDR2 loops of the TCR first weakly contact the MHC α-helices independent of the peptide and, subsequently, peptide contacts dominate stabilization and the peptide bound to MHC is recognized primarily by the hypervariable complementarity determining region 3 (CDR3) loops (22). Apparently, the TCR CDR3 regions have been shown to play a leading role in antigen binding and specificity. Several studies on alanine substitutions of TCR CDR3 residues have widely studied to determine the role of individual CDR3 residues on TCR affinity and functional avidity (23, 24). However, Rosenberg group has demonstrated that the TCR β-chain plays a more important role in antitumor response than the TCR α-chain by comparing the functional activity of AIB (α-IRES-β) and BIA (β-IRES-α) TCRs-modified PBL (25). It is universally acknowledged that the αβ TCR is assembled by a process of somatic gene rearrangement of variable (V), diversity (D), joining (J), and constant (C) gene fragments. The process includes stochastic N-nucleotide insertions and deletions at VDJ junctions *via* VNDNJ in β chain. Even the V(D)J junctions are to a large extent shared between different T cell clones (26). Therefore, in this article, alanine mutagenesis of the NDN region which is the most random and specific within the CDR3β of the bispecific TCR was firstly performed.

We have previously successfully identified that a bispecific TCR screened out from peripheral blood mononuclear cells (PBMCs) of a HLA-A*0201^+^ healthy individual using the CDR3 spectratype analysis recognizes both MTB Ag85B_199–207_ and HIV-1 Env_120–128_ peptide (27). However, it has not been known how residues on CDR3 loops contribute to the bispecific TCR contact with the peptide–MHC complexes. In this report, we provided an extensive investigation of residues in the predicted CDR3β of the bispecific TCR using single AA substitutions. Our data showed that three of the five substituted residues in CDR3β of the bispecific TCR caused a markedly diminished T cell response, whereas the remaining two alanine substitutions resulted in completely eliminated antigen response. These findings will provide an imperative foundation for generating the engineered high-affinity bispecific TCR for use in T cell adoptive immunotherapy for MTB/HIV coinfected individuals.

## Materials and Methods

### Cell Lines and Primary Cells

The 293T human embryonic kidney cells (ATCC CRL-11268) used for lentiviral production were cultured in Dulbecco’s modified Eagle’s medium (Corning, NY, USA) supplemented with 10% fetal bovine serum (FBS; Corning), 1% GlutaMAX-I (Thermo Fisher Scientific Inc., MA, USA), and 1% Minimum Essential Medium Non-Essential Amino Acids (Thermo Fisher Scientific Inc.). T2, which is a lymphoblastoid cell line deficient in TAP function, whose HLA-A*0201 molecules can be easily loaded with exogenous peptides, was grown in Iscove’s modified Dulbecco’s medium (Corning) contained with 20% FBS. The J.RT3-T3.5 cell line (kindly provided by Dr. Wei He, Peking Union Medical College, Beijing, China), which is a derivative mutant of the Jurkat leukemia cell line lacking surface expression of TCR α/β heterodimer and CD3 due to a defect in the TCR β-chain, was maintained in 10% FBS RPMI-1640 (Corning).

Peripheral blood mononuclear cells were isolated from blood of a HLA-A*0201 healthy donor with informed consent by Ficoll-Hypaque (Axis-Shield Diagnostics Ltd., Dundee, Scotland, UK) density gradient centrifugation. The research had been carried out in accordance with the World Medical Association Declaration of Helsinki and was approved by the ethics committee of the Southern Medical University. Monocyte-derived dendritic cells (DCs) were induced from the autologous PBMCs as previously described (28). CD8^+^ T cells were sorted from PBMCs using anti-CD8-labeled MACS magnetic beads (Miltenyi Biotec, Bergisch Gladbach, Germany) and were activated for 3 days by anti-CD3 (1 µg/ml), anti-CD28 (1 µg/ml) mAbs (BD Pharmingen, San Jose, CA, USA), and 100 U/ml interleukin-2 (IL-2; PeproTech, Rocky Hill, NJ, USA). Purified cells were then grown in RPMI-1640 medium contained with 10% FBS and 100 U/ml IL-2. All cells were cultured in a 37°C and 5% CO_2_ incubator.

### Generation of Lentiviral Vectors Encoding Wild-type (WT) and Variant TCRs

**Figure 1:**
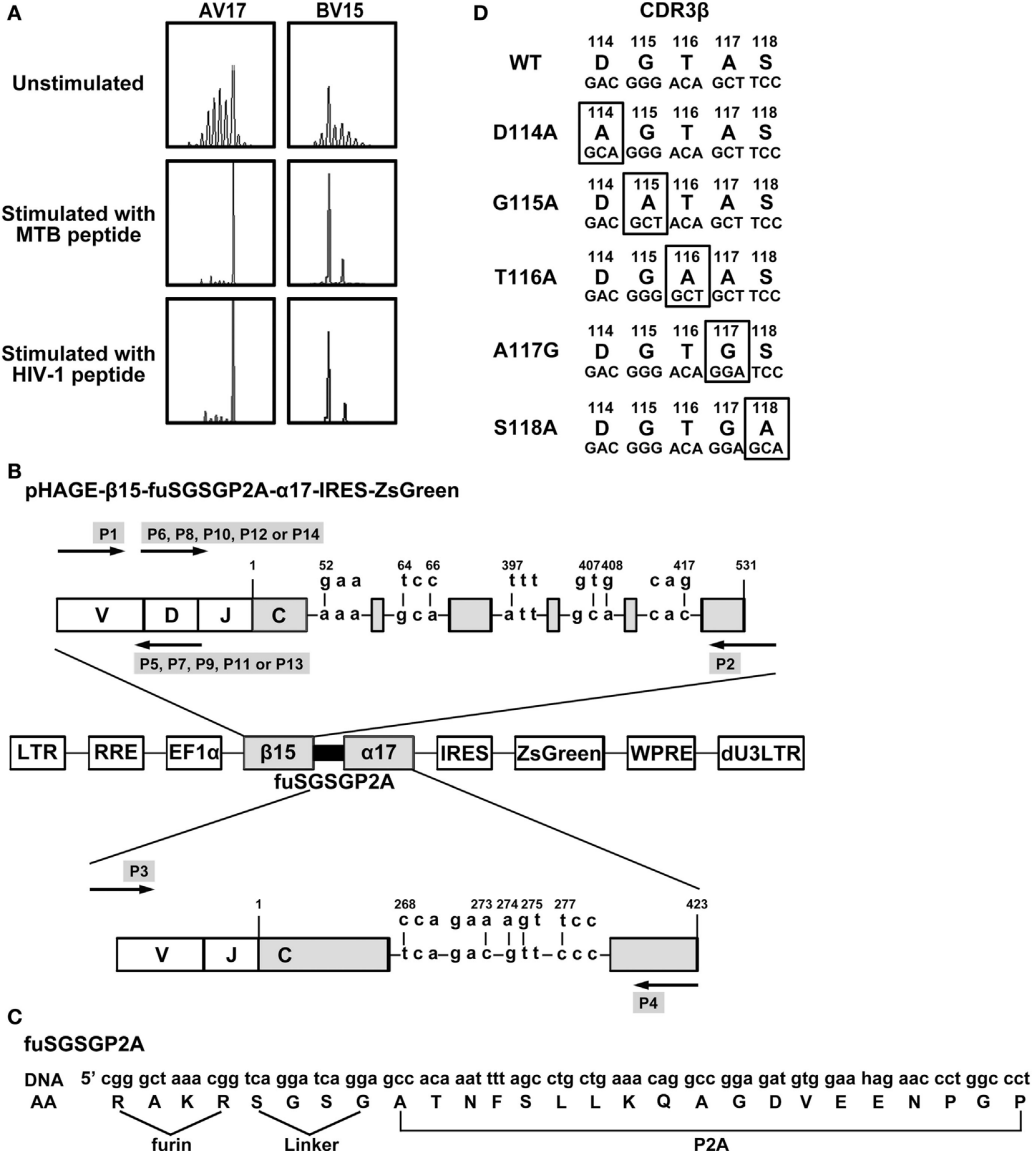
Construction of lentiviral vectors expressing WT and variant bispecific T cell receptors (TCRs). **(A)** Complementarity determining region 3 (CDR3) spectratypes of TCR Vα17 and Vβ15 gene families of CD8^+^ T cells before and after MTB Ag85B_199–207_ (KLVANNTRL) peptide or HIV-1 Env_120–128_ (KLTPLCVTL) peptide stimulation. **(B)** Schematic illustration of the lentiviral vector encoding WT or variant bispecific TCRs. TCR β- and α-chains were linked with furin-SGSG-P2A. Nine critical AAs in the C regions of β15 and α17 were replaced by their murine counterparts. **(C)** Details of furin-SGSG-P2A sequences. **(D)** Alanine substitutions at the CDR3 region of the bispecific TCR β-chain. AV, TCR Vα-chain; BV, TCR Vβ-chain; DNA: the DNA sequence; AA: the amino acid sequence; WT: wild-type.

The MTB Ag85B_199–207_ and HIV-1 Env_120–128_ bispecific TCR α17 and β15 genes identified by TCR CDR3 spectratype analysis (Figure 1A) as described in our previous study (27) were amplified from the plasmid pMX-β15-P2A-α17-IRES-GFP by PCR. Nine AAs in the C regions were replaced by murine counterparts as described previously (27). TCR α- and β-chains were linked with furin (RAKR)-linker (SGSG)-P2A ribosomal skip peptide sequence (29) by recombinant PCR. To increase translation efficiency, a Kozak consensus sequence (27) was included around the Vα ATG.

Briefly, to achieve the WT TCR β15-fuSGSGP2A-α17 gene, the plasmid pMX-β15-P2A-α17-IRES-GFP was first used as the template. The forward primer P1 and the reverse primer P2 (containing 5′-end of fuSGSGP2A) were used to generate the β-chain containing 5′-end of fuSGSGP2A (hereinafter referred to as S1). The primers P3 (containing 3′-end of fuSGSGP2A) and P4 were used to generate the α-chain containing 3′-end of fuSGSGP2A (fragment S2). Using S1 and S2 as the templates, the WT β15-fuSGSGP2A-α17 fragment was amplified using P1 and P4 primers. For generating the variant TCR β-chains, the 5′-end β15 fragment was amplified from the WT β15-fuSGSGP2A-α17 construct using the forward primer P1 and the reverse primer P5, P7, P9, P11or P13, and the 3′-end β15-fuSGSGP2A-α17 fragment was generated using the forward primer P6, P8, P10, P12, or P14 and the reverse primer P4. The corresponding fragments were then mixed and joined by carrying out a PCR using P1 and P4 primers to produce the variant TCR genes. The DNA fragments containing the WT and variant TCRs were then digested and inserted into the pHAGE-fullEF1a-MCS-IZsGreen lentiviral vector at XbaI and BamHI sites. All of the primers (P1–P14) are outlined in Table 1.

**Table 1:** Primers used for amplification of WT and variant T cell receptors.

Primer	Sequence (5′–3′)
P1	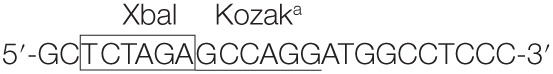
P2	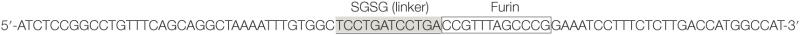
P3	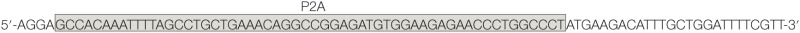
P4	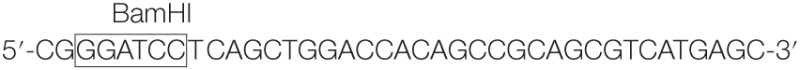
P5	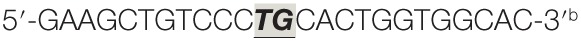
P6	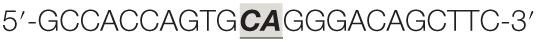
P7	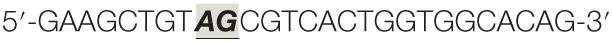
P8	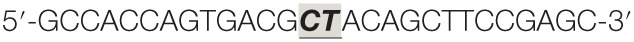
P9	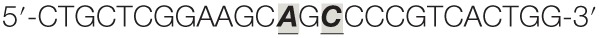
P10	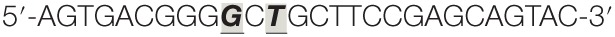
P11	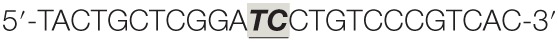
P12	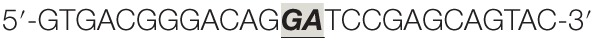
P13	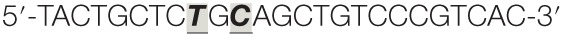
P14	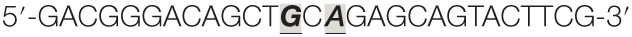
GAPDH forward	5′-GGATATTGTTGCCATCAATGACC-3′
GAPDH reverse	5′-AGCCTTCTCCATGGTGGTGAAGA-3′
β15-fuSGSGP2A-α17 forward	5′-TCCTGTCTGCCACCATCCTCTAT-3′
β15-fuSGSGP2A-α17 reverse	5′-CAGCCACAAAAACAGGAACGA-3′

*^a^Kozak sequence, a nucleotide sequence located in the 5′ untranslated mRNA region that allows ribosomes to recognize the initiator codon, was underlined*.

*^b^Mutated nucleotides were labeled underlined, bold, tilted, and shadowed simultaneously*.

### Production of Recombinant Lentiviral Particles

The 293T human embryonic kidney cells were passaged the day before infection to achieve 70–80% confluence at infection. Cells were transfected with the lentivirus triad of plasmids pHAGE, psPAX2, and pMD2.G using X-tremeGENE HP DNA Transfection Reagent (Roche, Mannheim, Germany) following the manufacturer’s instructions. Viral supernatants were harvested 48–72 h later. After filtering through a 0.45 µm filter (Millipore, Bedford, MA, USA), the supernatants were concentrated by ultracentrifugation at 50,000 *g*, 4°C, for 90 min. The recombinant lentiviral particles were recovered and aliquots were sharp-frozen at −80°C. Viral titers were evaluated on 293T cells by adding serial dilutions of concentrated virus suspension to 1 × 10^5^ cells in a 12-well plate (Nunc, Roskilde, Denmark) in the presence of polybrene (8 µg/ml; Sigma-Aldrich, St. Louis, MO, USA). Cells were analyzed 3 days after transduction for GFP expression by flow cytometry. Transduction activity was expressed in transduction units.

### Genetic Modification of CD8^+^ T Cells

CD8^+^ T cells were plated at 1 × 10^6^ cells/ml in 6-well plates (Nunc) in 10% FBS RPMI-1640 medium supplemented with 100 U/ml IL-2, 1 µg/ml anti-CD3, and 1 µg/ml anti-CD28 mAbs 72 h before transduction. The concentrated lentivirus suspension containing 8 µg/ml polybrene was added and after 4 h incubation, the fresh medium was supplemented to dilute the polybrene to 2 µg/ml. The procedure was repeated for an additional 2 days (total of three transductions). Five days after the third transduction (total of 8 days for infection), gene-modified CD8^+^ T cells were collected to eliminate the dead cells using the Dead Cell Removal Kit (Miltenyi Biotec) and detect the expression of GFP and incubated with APC-Cy7-anti-CD8 (Biolegend, San Diego, CA, USA), PE-labeled Ag85B_199–207_/HLA-A*0201 dextramer (Immudex, Copenhagen, Denmark) and APC-labeled Env_120–128_/HLA-A*0201 dextramer (Immudex) according to the manufacturer’s instructions to detected the exogenous TCRs by flow cytometry. In addition, RNA was extracted from transduced CD8^+^ T cells using E.Z.N.A.^®^ Total RNA Kit (OMEGA Biotek, Inc., Norcross, GA, USA), and reverse transcription was performed using RevertAid™ First Strand cDNA Synthesis Kit (Thermo Fisher Scientific, Inc.). Transcription of exogenous TCR genes was verified by PCR. Primer sequences used to amplify a portion of GAPDH and β15-fuSGSGP2A-α17 from human CD8^+^ T cells are summarized in Table 1.

### Cytokine Release Assays

T2 cells pulsed with HLA-A*0201-restricted MTB Ag85B_199–207_ peptide (KLVANNTRL), HIV-1 Env_120–128_ peptide (KLTPLCVTL), or CMV pp65_495–503_ peptide (NLVPMVATV) (10 µg/ml, or as described in figure legends; Proimmune, Oxford, UK) for 3 h at 37°C were incubated with CD8^+^ T cells. If IL-2 was the cytokine measured, the cells were washed with media without IL-2 prior to coculture. For these assays, 1 × 10^5^ effector cells (CD8^+^ T cells) and 1 × 10^5^ stimulator cells (T2 cells) were incubated in individual wells of 96-well *U*-bottom plate (Nunc) with a total volume of 0.2 ml for 24 h, except interferon-γ (IFN-γ), which was measured after 18 h of incubation. In some groups, DCs were transfected with the pV1J.ns-tPA-Ag85B plasmid (gifted by Dr. Kris Huygen in Pasteur Institute of Brussels, Brussels, Belgium) or the pCAGGS-Env plasmid (gifted by Dr. James M. Binley in Torrey Pines Institute for Molecular Studies, San Diego, CA, USA), respectively, using Lipofectamine 2000 Transduction Reagent (Invitrogen, Carlsbad, CA, USA).Cytokines were measured using IFN-γ, tumor necrosis factor-α (TNF-α), IL-2, granulocyte-macrophage colony-stimulating factor (GM-CSF) ELISA kits (ExCell Bio, ShangHai, China), and granzyme B (GrB) ELISA Kit (eBioscience, San Diego, CA, USA) according to the manufacture’s protocols.

### T Cell Proliferation Assays

T-cell proliferation was assessed using Cell Counting Kit-8 (CCK-8; Dojindo, Kumamoto, Japan) according to the manufacturer’s instructions. Briefly, 5 × 10^4^ CD8^+^ T cells were cocultured with 5 × 10^4^ peptide pulsed T2 cells in a 96-well *U*-bottom plate. After incubating for 2, 4, or 7 days, 100 µl/well of cell suspension was transferred into empty wells and 10 µl/well CCK-8 solutions were added. The plate was incubated at 37°C for 4 h, and the absorbance at 450 nm was measured using a spectral scanning multimode reader (Thermo Scientific Varioskan Flash, Thermo Fisher Scientific, Inc.).

### Intracellular Cytokine Staining

The genetically engineered T cells were also identified by flow cytometric analysis to assess intracellular IFN-γ production. Briefly, TCR gene-modified CD8^+^ T cells were stimulated by Ag85B_199–207_, Env_120–128_, or pp65_495–503_-loaded T2 cells in the presence of IL-2 (50 U/ml) for 18 h and then 10 µg/ml brefeldin A (Sigma-Aldrich) was added. After four additional hours of stimulation, 2 × 10^6^ of cocultured cells were stained with PE-labeled Ag85B_199–207_/HLA-A*0201 dextramer, APC-labeled Env_120–128_/HLA-A*0201 dextramer, and APC-Cy7-anti-CD8, fixed and permeabilized according to the manufacturer’s instructions (Cytofix/Cytoperm™ Fixation/Permeabilization Solution Kit, BD Pharmingen), followed by intracellular staining with PE-Cy7-anti-IFN-γ (eBioscience).

### EuTDA Cytotoxicity Assays

Cytotoxic activity of TCR-modified CD8^+^ T cells was determined using a DELFIA EuTDA cytotoxicity kit (Perkin-Elmer Life Sciences, Norwalk, CT, USA). Eu-labeled T2 cells or autologous DCs (5 × 10^3^) were incubated with transduced CD8^+^ T cells at the effector: target ratio of 30:1 (or as described in figure legends) for 4 h at 37°C. The plates were centrifugated and 20 µl of supernatants from each well were transferred to a fresh 96-well microtitration plate, which contained 200 µl of Eu Solution in each well. Fluorescence was detected using a Wallac Victor 2 Multilabel Counter (Perkin-Elmer Life Sciences, Norwalk, CT, USA). Percentage cytotoxicity was calculated by the following formula: [(experimental release − spontaneous release)/(maximum release − spontaneous release)] × 100.

### CD107a Degranulation Assays

In cytotoxic T cells, the cell surface mobilization of the CD107a was assessed as a marker for degranulation of lytic granules. In brief, 5 × 10^5^ gene-transduced CD8^+^ T cells were incubated with 5 × 10^5^ peptide-pulsed T2 cells for 4 h in the presence of 10 µg/ml brefeldin A and 5 µl PE-anti-CD107a (eBioscience). After the incubation, the cells were harvested, washed, and stained with antibody specific for CD8. Data analysis was performed by Flow cytometry.

### Expression of CD69 in Gene-Modified J.RT3-T3.5 Cells

J.RT3-T3.5 cells were seeded at a density of 5 × 10^5^ cells/ml in RPMI-1640 into 6-well plates before transduction and then incubated with concentrated lentivirus suspension. After 3 days of transduction, genetically engineered J.RT3-T3.5 cells were collected and cocultured with peptide-pulsed T2 cells. Eighteen hours later, the cocultured cells were collected, washed, and stained with APC-anti-CD69 (eBioscience) according to the manufacturer’s protocol. Data were analyzed by FACS.

### Statistical Analysis

All statistical analyses were performed using the SPSS version 17.0 for windows (SPSS, Chicago, IL, USA). A one-way ANOVA and multiple comparisons tests (least significant difference or Dunnett’s T3) were used to compare the differences between the experimental groups. *P*-values were two-sided. Differences with *P* < 0.05 were considered significant.

## Results

### WT and Variant TCRs Vectors Construction

The MTB Ag85B_199–207_ and HIV-1 Env_120–128_ bispecific TCR α17 and β15 genes were identified by TCR CDR3 spectratype analysis (Figure 1A) as described in our previous study (27). The lentiviral vector, pHAGE-β15-fuSGSGP2A-α17-IRES-IZsGreen, was constructed incorporating genes encoding β- and α-chains joined by furin (RAKR)-linker (SGSG)-P2A peptide sequence (Figures 1B,C). In this case, P2A was used to ensure equimolar production of β- and α-chain through a ribosomal skip mechanism. Addition of amino-acid linker (SGSG) before the P2A sequence could efficiently synthesize functionally active TCR. A furin cleavage site, which could be recognized in lymphocytes and facilitate removal of residual P2A peptide from the β-chain at the posttranslational level was incorporated at the 5′-end of the linker. To promote preferential pairing and improve cell surface expression of the exogenous TCR, nine critical AAs in the TCR α and β C regions were replaced by murine counterparts as described previously (27).

The AA residues 114–118 (DGTAS) of the TCR CDR3β were targeted for substitution (Figure 1D). Aspartic acid, glycine, threonine, and serine at positions 114, 115, 116, and 118, respectively, were substituted with alanine (designated D114A, G115A, T116A, and S118A, respectively), and alanine at position 117 were replaced with glycine (designated A117G). Using the primers listed in Table 1, these constructs containing single AA substitution were produced.

### TCR Expression of Transduced T Cells

**Figure 2:**
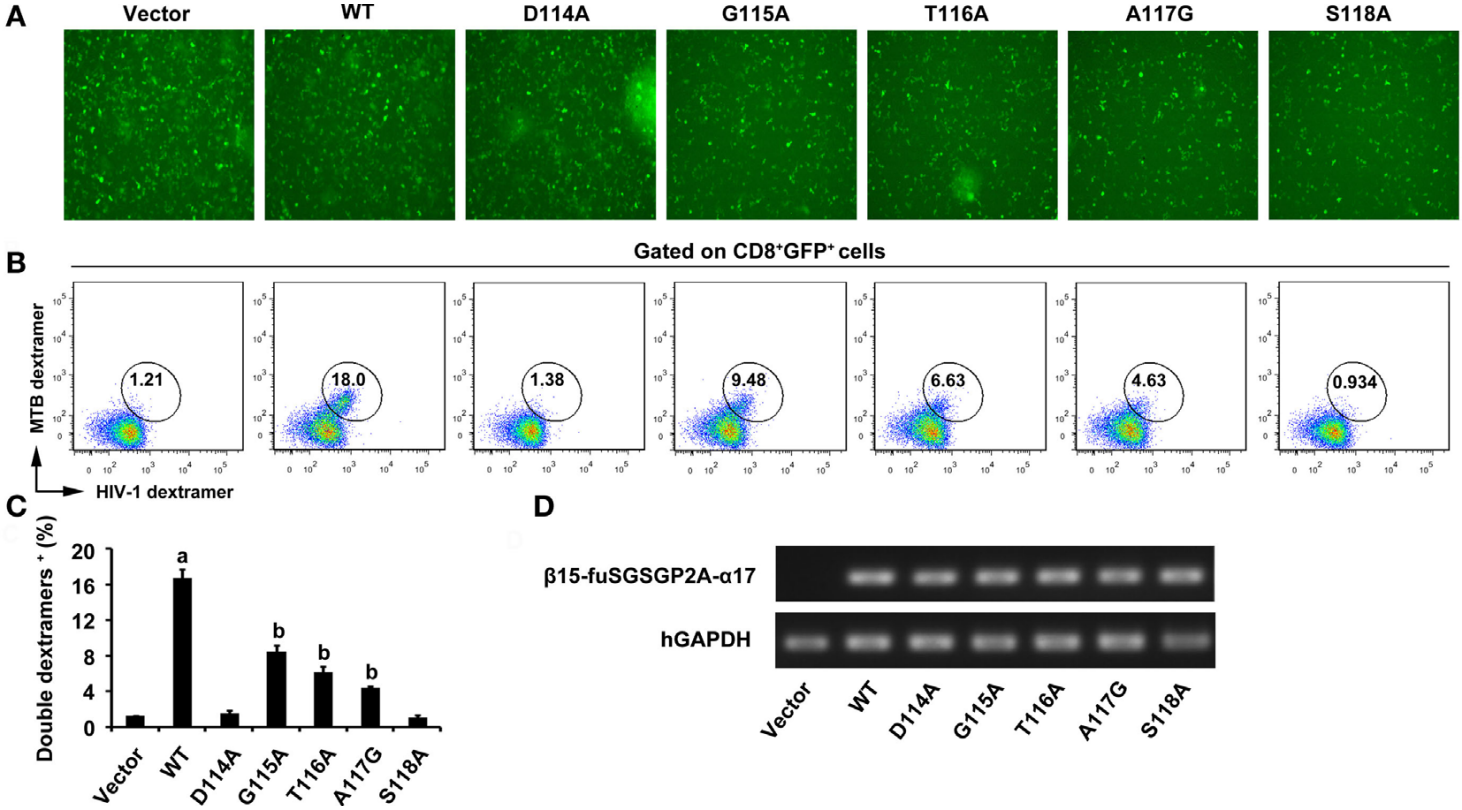
Expression of WT and variant bispecific T cell receptors (TCRs) after transduction of TCR genes into CD8^+^ T cells. **(A)** GFP expression was observed under fluorescence microscope. **(B)** Flow cytometric analysis of CD8^+^ T cells 5 days after the third lentiviral transduction. Cells were stained with APC-Cy7-anti-CD8, PE-Ag85B_199–207_/HLA-A*0201 dextramer, and APC-Env_120–128_/HLA-A*0201 dextramer. The data were analyzed within the GFP-positive population. **(C)** Shown is the histogram analyzed for the FACS scatter plots. **(D)** The PCR products of a segment of β15-fuSGSGP2A-α17 were analyzed on an agarose gel. ^a^*P* < 0.05 compared to other six groups. ^b^*P* < 0.05 compared to vector. Vector, transduced with empty vector only carrying the GFP gene; MTB dextramer: PE-Ag85B_199–207_/HLA-A*0201 dextramer; HIV-1 dextramer: APC-Env_120–128_/HLA-A*0201 dextramer.

After 5 days of the third lentiviral transduction, green fluorescence was clearly observed in empty vector transduced cultures and the WT and variant TCRs gene-modified CD8^+^ T cells (Figure 2A). As there is no commercial anti-Vα17 or Vβ15 antibody, MHC dextramers were used to determine the expression of transduced TCRs and assess TCRs affinity. Measurement by flow cytometry showed that about 16% of the CD8^+^ T cells were double-positive for the Ag85B_199–207_/HLA-A*0201 dextramer and Env_120–128_/HLA-A*0201 dextramer within the GFP-positive population in the WT TCR-transduced cultures, which was significantly higher than other groups (*P* < 0.05). Similar percentage levels of double-dextramers staining were observed on CD8^+^ T cells transduced with the G115A, T116A, and A117G TCRs, which showed a significant increase compared with empty vector transduced T cells, respectively (*P* < 0.05). Significant difference of the level of double-dextramers binding was not observed between CD8^+^ T cells transduced with the D114A TCR construct and empty vector transduced T cells (*P* > 0.05), as well as the S118A TCR transductant (Figures 2B,C). Transcription of transduced TCR genes was further verified by PCR. The electrophoretic band of a segment of β15-fuSGSGP2A-α17 was clearly observed in the WT and variant TCRs transductants, while it was not observed in the empty vector transduced cells (Figure 2D).

### Cytokine Release in the WT and Variant TCRs Transductants

**Figure 3:**
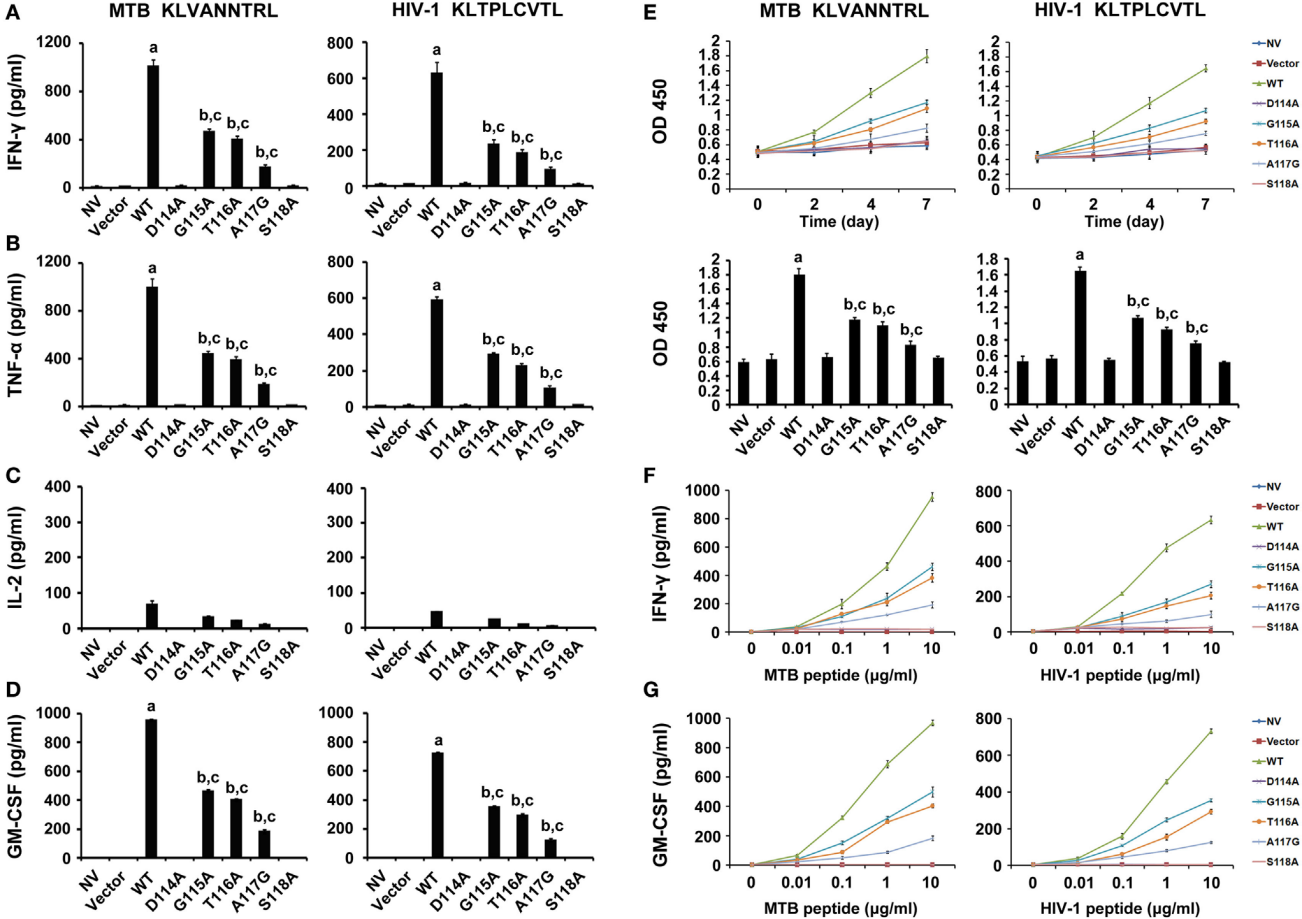
Cytokine secretion and proliferation of WT and variant T cell receptors gene-modified CD8^+^ T cells in response to antigen peptides stimulation. **(A–D)** CD8^+^ T cells were cocultured with T2 loaded with 10 µg/ml of MTB Ag85B_199–207_ (KLVANNTRL) (left panel) or HIV-1 Env_120–128_ (KLTPLCVTL) (right panel) and were then tested for their ability to release interferon-γ (IFN-γ), tumor necrosis factor-α (TNF-α), interleukin-2, or GM-CSF. **(E)** Proliferation of CD8^+^ T cells after coculture with peptide-pulsed T2 cells measured by cell counting kit-8. **(F,G)** Sensitivity of IFN-γ or GM-CSF secretion to dilutions of MTB or HIV-1 peptide. Values indicate average counts of three independent experiments ±SD. ^a^*P* < 0.05 compared to other seven groups. ^b^*P* < 0.05 compared to NV. ^c^*P* < 0.05 compared to Vector. NV: no vector. MTB peptide: Ag85B_199–207_ peptide; HIV-1 peptide: Env_120–128_ peptide.

To evaluate the function of the bispecific TCR CDR3β variants in CD8^+^ T cells, the effector cytokines were measured. The WT TCR gene-modified CD8^+^ T cells stimulated by either MTB Ag85B_199–207_ peptide or HIV-1 Env_120–128_ peptide-pulsed T2 cells secreted significantly higher levels of IFN-γ than other groups (*P* < 0.05). Compared with untransduced or empty vector transduced T cells, the G115A, T116A, or A117G TCR transductant when cocultured with T2 cells loaded with MTB peptide or HIV-1 peptide showed a significant increase in IFN-γ secretion (*P* < 0.05), while the D114A and S118A TCR transductants did not (*P* > 0.05) (Figure 3A). Similarly, the WT TCR-transduced CD8^+^ T cells specifically produced significant levels of TNF-α when exposed to MTB peptide or HIV-1 peptide-pulsed T2 cells compared with other groups (*P* < 0.05). CD8^+^ T cells transduced with G115A, T116A, or A117G TCR variant when cocultured with MTB peptide or HIV-1 peptide-pulsed T2 cells made high amounts of TNF-α compared to untransduced or empty vector transduced T cells (*P* < 0.05), while the D114A and S118A TCR-transduced CD8^+^ T cells did not (*P* > 0.05) (Figure 3B). CD8^+^ T cells transduced with the WT, G115A, T116A, or A117G TCR secreted low levels of IL-2, although higher than the other four groups (Figure 3C). Similarly with IFN-γ and TNF-α, the amount of GM-CSF produced by the WT TCR engineered CD8^+^ T cells following exposure to T2 cells pulsed with MTB peptide or HIV-1 peptide was significantly higher than that in the other seven groups (*P* < 0.05). CD8^+^ T cells expressing the G115A, T116A, or A117G TCR secreted highly elevated levels of GM-CSF in response to MTB peptide or HIV-1 peptide-pulsed T2 cells compared to control groups (untransduced or empty vector transduced group, *P* < 0.05), while T cells expressing the D114A and S118A TCR failed to produce significant levels of GM-CSF (*P* > 0.05) (Figure 3D).

The relative reactivity of the WT and variant TCRs gene-engineered T cells was detected by incubating transduced CD8^+^ T cells with T2 cells pulsed with limiting dilutions of MTB peptide or HIV-1 peptide (Figures 3F,G). The WT TCR, as well as G115A, T116A, and A117G TCR-transduced T cells, recognized T2 cells pulsed with as little as 0.01 µg/ml MTB peptide or HIV-1 peptide. But cytokines secretion exerted a dramatic reduction in G115A, T116A, and A117G TCR-transduced T cells compared with those in the WT TCR-transduced cultures. In marked contrast, no significant differences in cytokines production were observed in untransduced, empty vector transduced, D114A and S118A TCR-transduced cell populations at every peptide concentration.

**Figure 4:**
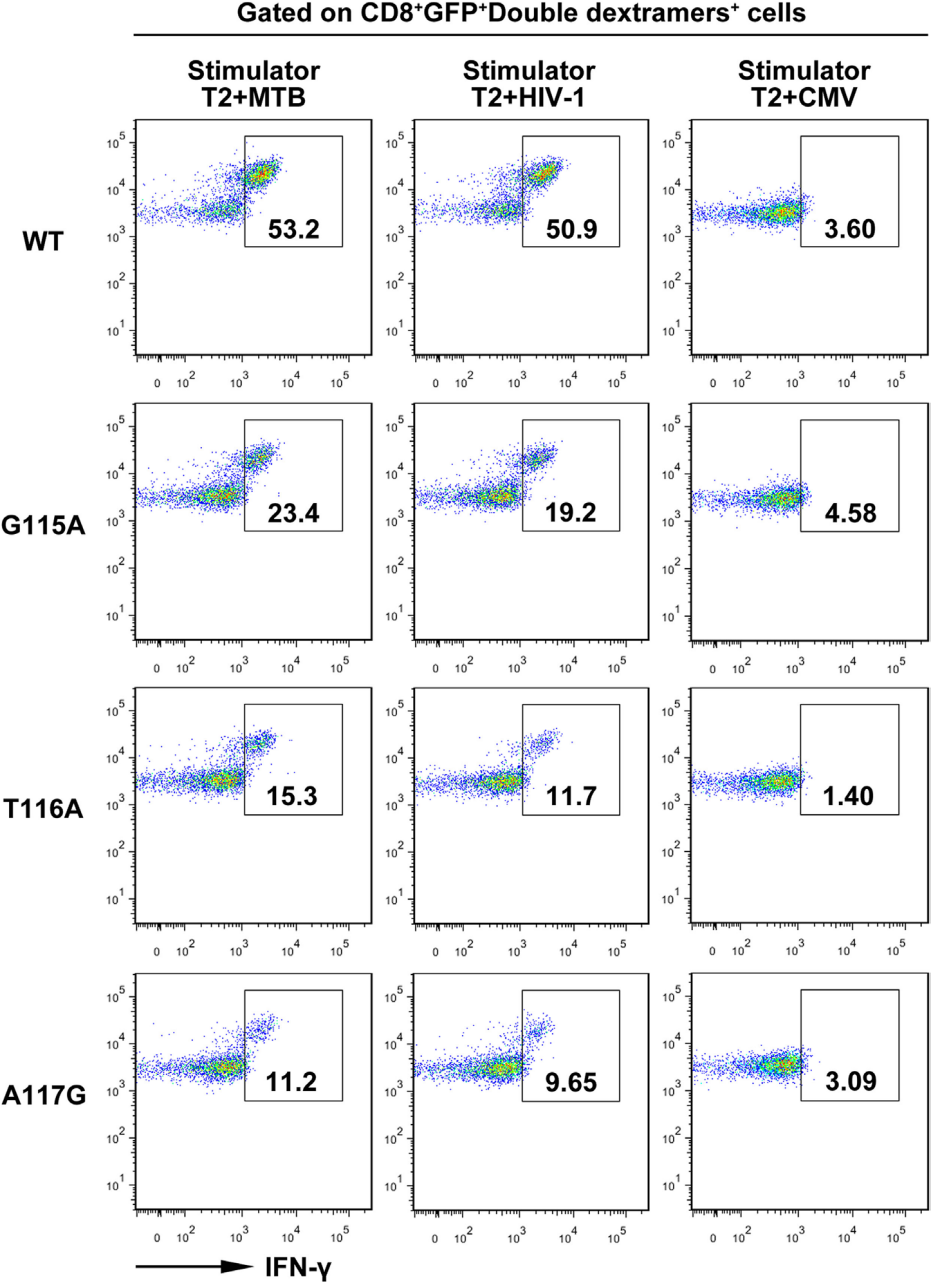
Intracellular staining of transduced CD8^+^ T cells for interferon-γ (IFN-γ) production. Shown are the resultant FACS scatter plots for CD8^+^ T cells transduced with WT, G115A, T116A, or A117G T cell receptor vector. Cells were cocultured with T2 pulsed with MTB Ag85B_199–207_, HIV-1 Env_120–128_, or control peptide CMV pp65_495–503_. Cells were gated for CD8^+^GFP^+^double dextramers^+^ cells and then analyzed for intracellular IFN-γ.

Furthermore, intracellular cytokine staining showed that about 53 and 50% of the WT TCR-engineered CD8^+^ T cells within the GFP^+^ double dextramers^+^ population were positive for intracellular IFN-γ in the presence of MTB peptide or HIV-1 peptide, respectively. In contrast, only a very low proportion of IFN-γ-producing T cells was observed in the control group following exposure to the irrelevant CMV pp65_495–503_ peptide-pulsed T2 cells. Compared with the WT TCR-transduced T cells, the G115A, T116A, or A117G TCR transductant when cocultured with T2 cells loaded with MTB peptide or HIV-1 peptide showed a significant decrease in IFN-γ secretion (Figure 4).

### Antigen-Specific Proliferation of the WT and Variant TCRs-Engineered CD8^+^ T Cells

To confirm whether these gene-modified cells would proliferate *in vitro*, the CCK-8 assay was performed (Figure 3E). The speed of proliferation of WT TCR engineered CD8^+^ T cells stimulated with MTB peptide or HIV-1 peptide-loaded T2 cells was significantly faster than that in other groups at 2, 4, and 7 days post-stimulation, particularly at day 7 (*P* < 0.05). CD8^+^ T cells transduced with G115A, T116A, and A117G TCR proliferated significantly greater than control groups after exposure to T2 cells loaded with MTB peptide or HIV-1 peptide, especially on day 7 (*P* < 0.05). As expected, no obvious differences in proliferation were observed in untransduced, empty vector transduced, D114A and S118A TCR-transduced cells at every time (*P* > 0.05).

### Cytolytic Activity of CD8^+^ T Cells Expressing the WT and Variant TCRs

**Figure 5:**
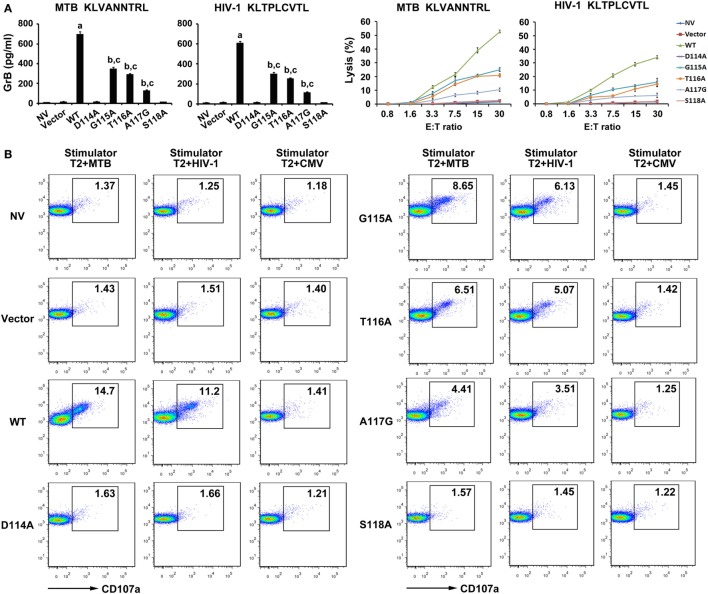
Cytolytic activity of WT and variant T cell receptors (TCRs) gene-modified CD8^+^ T cells in response to antigen peptides stimulation. CD8^+^ T cells were cocultured with T2 loaded with 10 µg/ml of MTB Ag85B_199–207_ peptide or HIV-1 Env_120–128_ peptide. **(A)** Secretion of granzyme B (GrB) by T cells was tested using ELISA, and the direct cytotoxicity on T2 cells was evaluated using DELFIA assay. **(B)** Expression of degranulation marker CD107a on WT and variant TCRs-transduced CD8^+^ T cells was detected by FACS analysis after coculture with T2 cells. ^a^*P* < 0.05 compared to other seven groups. ^b^*P* < 0.05 compared to NV. ^c^*P* < 0.05 compared to Vector. MTB: Ag85B_199–207_ peptide; HIV-1: Env_120–128_ peptide; CMV: pp65_495–503_ peptide.

To determine the lytic function of the transduced CD8^+^ T cells, the GrB release and EuTDA cytotoxicity assays were first carried out (Figure 5A). GrB secretion of the WT TCR gene-modified T cells were significantly higher than that in the other seven groups when exposed to T2 cells pulsed with MTB peptide (*P* < 0.05). CD8^+^ T cells expressing G115A, T116A, and A117G TCR when cocultured with MTB peptide-pulsed T2 cells had higher GrB secretion than control groups (*P* < 0.05), while the D114A and S118A TCR transductants did not (*P* > 0.05). Meanwhile, the WT TCR-transduced T cells readily lysed T2 cells pulsed with MTB peptide. But lysis exerted a significant reduction in G115A, T116A, and A117G TCR gene-modified T cells compared with those in the WT TCR-transduced cells. In contrast, there was little lysis observed in the untransduced, empty vector transduced, D114A and S118A TCR-transduced cells. When T cells exposed to T2 cells pulsed with HIV-1 peptide, the situations of GrB secretion and the percent-specific lysis were the same as those of MTB peptide.

As an additional test of CD8^+^ T cells cytotoxic activity, degranulation measured as cell surface mobilization of CD107a was next determined (Figure 5B). In this experiment, T cells transduced with the WT TCR when cocultured with MTB peptide or HIV-1 peptide-pulsed T2 cells showed a significantly enhanced expression of CD107a compared with the other seven groups. Consistent with the previous experiments, T cells expressing G115A, T116A, and A117G TCR following exposure to T2 cells loaded with MTB peptide or HIV-1 peptide produced significantly higher levels of CD107a than control groups, while the D114A and S118A TCR transductants produced an equivalent level of CD107a compared with untransduced or empty vector transduced group.

### Assessment of the Function of WT and Variant TCRs Gene-Modified CD8^+^ T Cells against Endogenous Peptides

**Figure 6:**
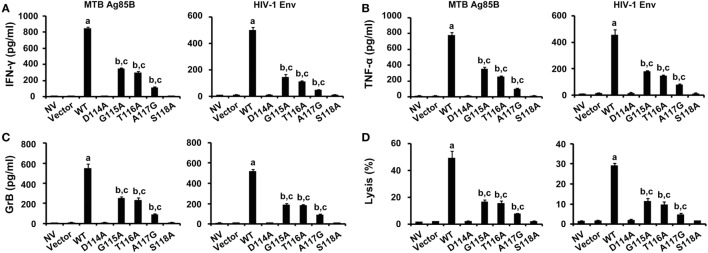
Immune responses to endogenous antigens by WT and variant T cell receptors-transduced CD8^+^ T cells. T cells were cocultured with dendritic cells transfected with the pV1J.ns-tPA-Ag85B plasmid or the pCAGGS-Env plasmid. Activities of T cells were analyzed by ELISA of interferon-γ (IFN-γ) **(A)**, tumor necrosis factor-α (TNF-α) **(B)**, granzyme B (GrB) **(C)**, and DELFIA assay **(D)**. MTB Ag85B: the Ag85B-expressing plasmid pV1J.ns-tPA-Ag85B; HIV-1 Env: the Env-expressing plasmid pCAGGS-Env. ^a^*P* < 0.05 compared to other seven groups. ^b^*P* < 0.05 compared to NV. ^c^*P* < 0.05 compared to Vector.

The abilities of CD8^+^ T cells expressing the WT and variant TCRs that recognize naturally processed epitopes from MTB and HIV presented by autologous DCs were examined (Figure 6). DCs transfected with the Ag85B-expressing plasmid pV1J.ns-tPA-Ag85B or the Env-expressing plasmid pCAGGS-Env were cocultured with TCR gene-modified CD8^+^ T cells. In agreement with expectations, the WT TCR engineered T cells responded to both Ag85B- and Env-expressing plasmids with significantly higher levels of cytokines secretion and lytic function when compared with the other seven groups (*P* < 0.05). Compared with untransduced or empty vector transduced group, T cells expressing G115A, T116A, and A117G TCR showed significantly elevated activities when exposed to the endogenously presented antigens by DCs (*P* < 0.05), while the D114A and S118A TCR transductants did not (*P* > 0.05).

### Expression of CD69 in Genetically Engineered J.RT3-T3.5 Cells

**Figure 7:**
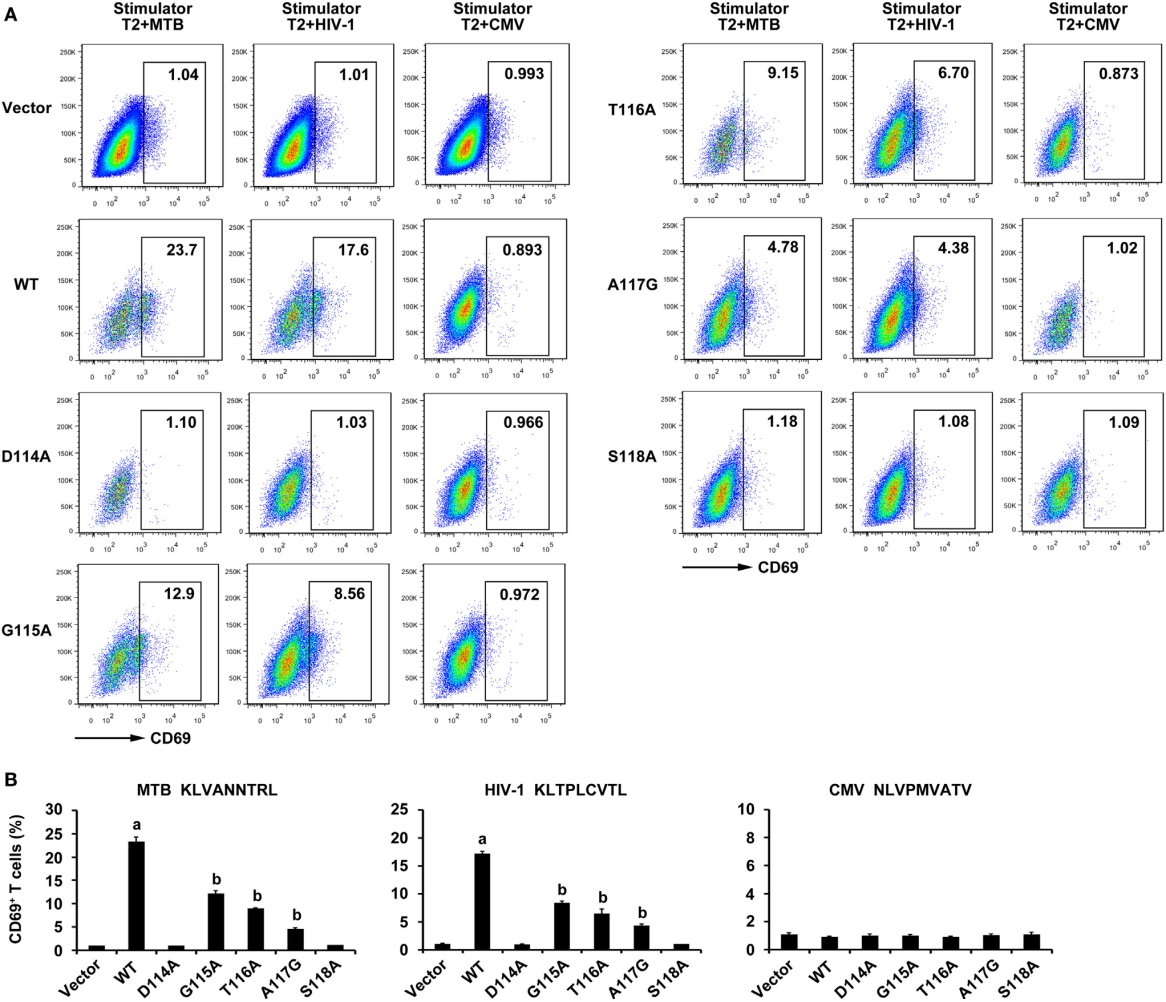
CD69 expression of WT and variant T cell receptor (TCR)-transduced J.RT3-T3.5 cells in response to antigen peptides stimulation. Cells were cocultured with T2 cells pulsed with Ag85B_199–207_, Env_120–128_, or control peptide pp65_495–503_. **(A)** Shown are the resultant FACS scatter plots for CD69 expression on J.RT3-T3.5 cells transduced with empty vector, WT, or variant TCRs vectors within the GFP-positive population. **(B)** Shown are the histograms analyzed for the FACS scatter plots. ^a^*P* < 0.05 compared to other six groups. ^b^*P* < 0.05 compared to Vector. MTB: Ag85B_199–207_ peptide; HIV-1: Env_120–128_ peptide; CMV: pp65_495–503_ peptide.

To further evaluate the properties of the WT and variant TCRs, constructs were transfected into the TCR negative T cell line J.RT3-T3.5. As might be expected, the expression of CD69, which is an immediate marker of early T cell activation in WT TCR-transduced J.RT3-T3.5 cells when incubated with T2 cells pulsed with MTB peptide or HIV-1 peptide showed a significant increase compared with those in the other six groups (*P* < 0.05). CD69 expression of the G115A, T116A, and A117G TCR-transduced J.RT3-T3.5 cells responded to both peptides exhibited a significant increase in contrast to that of empty vector transduced group (*P* < 0.05), while the CD69 expression of D114A and S118A TCR transductants did not (*P* > 0.05). Comparably, no significant increase in CD69 expression was observed in all groups after exposure to T2 cells pulsed with the irrelevant CMV pp65_495–503_ peptide (Figure 7).

## Discussion

In this study, we demonstrated the importance of AAs in the predicted CDR3 of the bispecific TCR β-chain in recognition of MTB Ag85B_199–207_ peptide and HIV-1 Env_120–128_ peptide. It is unlikely that the AA substitutions introduced into the bispecific TCR β3 loop disrupt the conformation of the entire TCR. As alanine, the smallest chiral AA, is one of the richest AAs and is both a buried or solvent-exposed residue in protein structures, replacing residues by it, which removes side chains beyond the β carbon, should be tolerated in loop structures (30). Actually, dextramer staining with MTB and HIV-1 dextramer demonstrated that the mutant TCRs exhibiting diminished antigen responses retained the peptides recognition by these dextramers (Figure 2B). This means that the overall conformations of the mutant TCRs were not disrupted. Of course, there are also some studies proving that alanine substitutions can cause conformational change in the Ig-like molecular CD4 as estimated by antibody binding (31, 32). The TCR residues in the C, C′, F, and G β-chains involved in the TCR α-β interface presumably have the strongest impact on chain pairing (33). However, we abstained from substituting at these AAs. As a matter of fact, mutant TCRs were effectively expressed in J.RT3-T3.5 cells, which lack surface expression of either CD3 or the TCR α/β heterodimer. The TCR/CD3 complexes expressed by the transductants were functional, as these cells were capable of expressing CD69 in response to MTB and HIV-1 peptide presented by T2 cells (Figure 7), proving that the AA substitutions in CDR3β disrupted neither association with TCR α chain or CD3 polypeptides, nor signal transduction through the TCR/CD3 complex. Thus, the most likely explanation for the diminished response by the mutant bispecific TCR transductants is that the alanine substitutions change the antigen-binding site on the TCR.

Amino acids in the predicted β3 loop of the bispecific TCR seem to be particularly crucial for recognition of peptide–MHC complexes. Mutation of Asp^114^ or Ser^118^ to alanine abolished the antigen response. In contrast, the other three residues in the β3 loop severely diminished but not abolished ligand recognition when mutated to alanine or glycine (G115A, T116A, A117G). The negatively charged Asp^114^ residue in the CDR3β of the bispecific TCR is required for TCR function. This could be due to the fact that Asp^114^ is likely to form a salt bridge with a positively charged residue or the positively charged antigen in peptide–MHC complex. A lot of antigen–antibody interfaces include one or more salt bridges (34). The S118A mutation, which removes a single side chain hydroxyl group, eliminated the antigen response, illustrating the significant impact that small structural changes can have on T cell recognition. The substitution of alanine for Thr^116^ gives rise to the removal of its methyl and hydroxyl groups, making the side chain relatively smaller. If Thr^116^ contacts the antigenic peptide, replacement of AAs on the antigenic peptide with bulkier side chains probably compensate for the substitution of alanine for Thr^116^. This hypothesis had been sufficiently tested in arsonate-conjugated peptide OVA (35).

As mentioned above, we identified the critical residues in the CDR3β of the bispecific TCR in the initial alanine substitution scan. On the basis of the results, single AA substitutions at these five critical residues will be synthesized to produce a 18 AA substitution library (except for the AA at each position and alanine or glycine) at each position by site-directed mutagenesis. Within these AA variants, the high-affinity TCR, which is likely to enhance antigen-specific reactivity in TCR gene-modified T cells may be identified. A study of the class II MHC molecular I-A^d^-restricted D5 TCR, which specifically recognizes arsonate-conjugated peptides, led to the identification of alanine substitutions in TCR CDRs that diminished T cell recognition, as well as substitutions that had no obvious effect, but failed to exhibit substitutions that enhanced T cell function (36). However, site-directed mutagenesis of the CDR regions of the murine 2C TCR that recognizes the H-2K^b^-restricted peptide as well as the allo-MHC molecule H-2L^d^-restricted peptide was performed to identify the high-affinity variants that in the meantime seemed to sustain specificity for the cognate peptide (37). A high-affinity mutant 2C TCR possessing affinity that was 300-fold higher than the WT TCR significantly increased the response of a CD8-negative T cell line that expressed the mutant TCR to peptide-loaded target cells (38). Furthermore, AA substitutions in the 1G4 TCR CDR regions, which had higher affinities than the native TCR not only markedly enhanced the ability of CD8^+^ T cells to recognize the NY-ES0–1/HLA-A*02 complex but also simultaneously showed enhanced antigen-specific reactivity in CD4^+^ T cells (39).

It has been reported that the exceedingly narrow window of native TCR-peptide-binding affinities, which distinguish between thymic positive and negative selection restricts the activity of the T cells, and T cell activity seems to be connected with the effective clearance of tumor cells and infected cells *in vivo* (40). Thus, genetically modified T cells expressing high-affinity TCRs against disease-specific antigens probably have the superior efficacy for adoptive immunotherapy. Carpenter et al. have reported that the primary response mediated by the immunodominant MTB antigen TB10.4-specific CD8^+^ T cells possessing high-affinity TCR outnumbers the memory response mediated by T cells expressing decreased affinity TCR during MTB challenge in mice (41). *Ex vivo* polyclonal CD8^+^ T cells efficiently redirected by a high-affinity HIV Gag-specific TCR eliminated CD4^+^ T cells from HLA-A*0201^+^ antiretroviral therapy-treated patients after reactivation of inducible HIV *in vitro* (12). Another well-documented example is that PBL-derived T cells genetically engineered with a high-affinity, HLA-A2-restricted, hepatitis C virus (HCV) NS3-reactive TCR can recognize naturally processed antigen and elicit CD8-independent recognition of both peptide-loaded targets and HCV^+^ human hepatocellular carcinoma cell lines. Furthermore, these cells can mediate regression of established HCV-associated hepatocellular carcinoma *in vivo* (11). However, genetically engineered T cells expressing high-affinity TCRs for the treatment of MTB/HIV coinfection have not been reported.

Despite our results have fully proved the importance of the AAs in the predicted CDR3 of the bispecific TCR β-chain in recognition of the peptide–MHC complexes, we cannot currently exclude the possibility that other regions of the TCR help antigen binding. For example, we should further study the function of the AAs in the CDR1β, CDR2β, CDR1α, CDR2α, CDR3α, as well as the “fourth” hypervariable loop of the bispecific TCR, which has been established the importance of involving in T cell recognition by a CD1d-restricted TCR (42).

In conclusion, our experiments indicated that three of the five substituted residues in CDR3β of the bispecific TCR significantly decreased T cell response, whereas the other two substituted residues completely abolished the antigen response. These results will provide an imperative foundation for generating the improved high-affinity bispecific TCR for use in T cell adoptive immunotherapy for MTB/HIV coinfected patients.

## Ethics Statement

This study was carried out in accordance with the recommendations of the International Committee of Medical Journal Editors and the ethics committee of the Southern Medical University with written informed consent from all subjects. All subjects gave written informed consent in accordance with the Declaration of Helsinki. The protocol was approved by the ethics committee of the Southern Medical University.

## Author Contributions

C-YZ participated in the cell experiments, substantially contributed to molecular biology studies and immunoassays, performed the statistical analysis, and drafted the manuscript. R-NW participated in interpretation of data and molecular biology studies. QW participated in research design, cell and virus infection experiments, and molecular biology studies. W-TH and S-MZ participated in cell and virus infection experiments. X-LD and J-HY participated in cell culture, immunoassay, and interpretation of data. LM conceived of the study and participated in its design and coordination and revised the manuscript critically. All authors read and approved the final manuscript.

## Conflict of Interest Statement

The authors declare that the research was conducted in the absence of any commercial or financial relationships that could be construed as a potential conflict of interest. The reviewer, IO, and handling editor declared their shared affiliation and the handling editor states that the process nevertheless met the standards of a fair and objective review.

## Abbreviations

MTB, *Mycobacterium tuberculosis*; HIV, human immunodeficiency virus; TCR, T cell receptor; CDR3, complementarity determining region 3; MHC, major histocompatibility complex; β, beta; TB, tuberculosis; V, variable; D, diversity; J, joining; C, constant; PBMCs, peripheral blood mononuclear cells; FBS, fetal bovine serum; DCs, dendritic cells; IL-2, interleukin-2; WT, wild-type; AAs, amino acids; IFN-γ, interferon-γ; TNF-α, tumor necrosis factor-α; GM-CSF, granulocyte-macrophage colony-stimulating factor; GrB, granzyme B; CCK-8, cell counting kit-8.
